# Crystal structure of 4-[(*E*)-(4-fluoro­benzyl­idene)amino]-3-methyl-1*H*-1,2,4-triazole-5(4*H*)-thione

**DOI:** 10.1107/S2056989015020125

**Published:** 2015-11-04

**Authors:** P. S. Manjula, B. K. Sarojini, B. Narayana, K. Byrappa, S. Madan Kumar

**Affiliations:** a Department of Chemistry, P A College of Engineering, Nadupadavu 574 153, D.K., Mangaluru, India; b Department of Industrial Chemistry, Mangalagangotri, Mangalore University, Mangaluru 574 199, India; c Department of Chemistry, Mangalagangotri, Mangalore University, Mangaluru 574 199, India; d Department of Materials Science, Mangalagangotri, Mangalore University, Mangaluru 574 199, India; e PURSE Lab, Mangalagangotri, Mangalore University, Mangaluru 574 199, India

**Keywords:** crystal structure, 1,2,4-triazole-5(4*H*)-thione, fluoro­benzene, C—H⋯S hydrogen bond, N—H⋯S hydrogen bond, π–π stacking contacts

## Abstract

The title compound, C_10_H_9_FN_4_S, crystallizes with two mol­ecules (*A* and *B*) in the asymmetric unit. The dihedral angle between the planes of the trizole and fluoro­benzene rings is 7.3 (3)° in mol­ecule *A* and 41.1 (3)° in mol­ecule *B*. Mol­ecule *A* features an intra­molecular C—H⋯S hydrogen bond, which closes an *S*(6) ring. In the crystal, *A*+*B* dimers linked by pairs of N—H⋯S hydrogen bonds occur, generating *R*_2_^2^(8) loops. Weak π–π stacking contacts [centroid–centroid separation = 3.739 (6) Å] are also observed.

## Related literature   

For a related structure, see: Manjula *et al.* (2015 ▸).
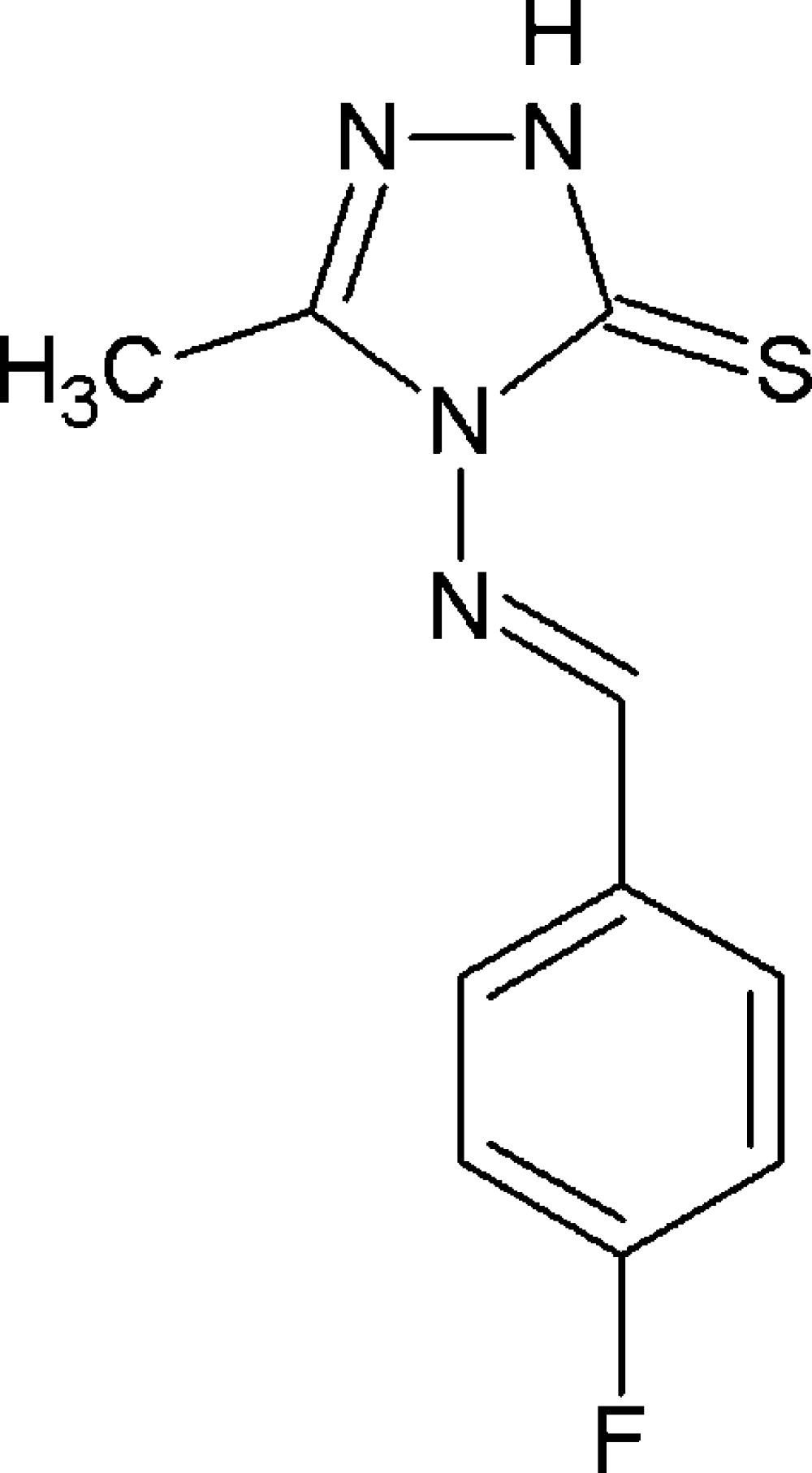


## Experimental   

### Crystal data  

C_10_H_9_FN_4_S
*M* *_r_* = 236.27
Triclinic, 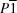

*a* = 9.1878 (13) Å
*b* = 11.0083 (16) Å
*c* = 12.9851 (18) Å
α = 99.526 (6)°
β = 104.963 (13)°
γ = 113.202 (19)°
*V* = 1112.1 (3) Å^3^
*Z* = 4
Mo *K*α radiation
μ = 0.28 mm^−1^
*T* = 273 K
0.46 × 0.27 × 0.14 mm

### Data collection  

Rigaku Saturn724+ diffractometer
Absorption correction: numerical (*NUMABS*; Rigaku 1999 ▸) *T*_min_ = 0.913, *T*_max_ = 0.961
7140 measured reflections
3864 independent reflections
2129 reflections with *I* > 2σ(*I*)
*R*_int_ = 0.042

### Refinement  

*R*[*F* ^2^ > 2σ(*F* ^2^)] = 0.068
*wR*(*F* ^2^) = 0.241
*S* = 1.14
3864 reflections
291 parameters
H-atom parameters constrained
Δρ_max_ = 0.38 e Å^−3^
Δρ_min_ = −0.39 e Å^−3^

### 

Data collection: *CrystalClear SM Expert* (Rigaku, 2011 ▸); cell refinement: *CrystalClear SM Expert*; data reduction: *CrystalClear SM Expert*; program(s) used to solve structure: *SHELXS97* (Sheldrick, 2008 ▸); program(s) used to refine structure: *SHELXL2014* (Sheldrick, 2015 ▸); molecular graphics: *Mercury* (Macrae *et al.*, 2008 ▸); software used to prepare material for publication: *OLEX2 *(Dolomanov *et al.*, 2009 ▸).

## Supplementary Material

Crystal structure: contains datablock(s) I. DOI: 10.1107/S2056989015020125/hb7528sup1.cif

Structure factors: contains datablock(s) I. DOI: 10.1107/S2056989015020125/hb7528Isup2.hkl

Supporting information file. DOI: 10.1107/S2056989015020125/hb7528Isup3.cml

**Fig. 1.**
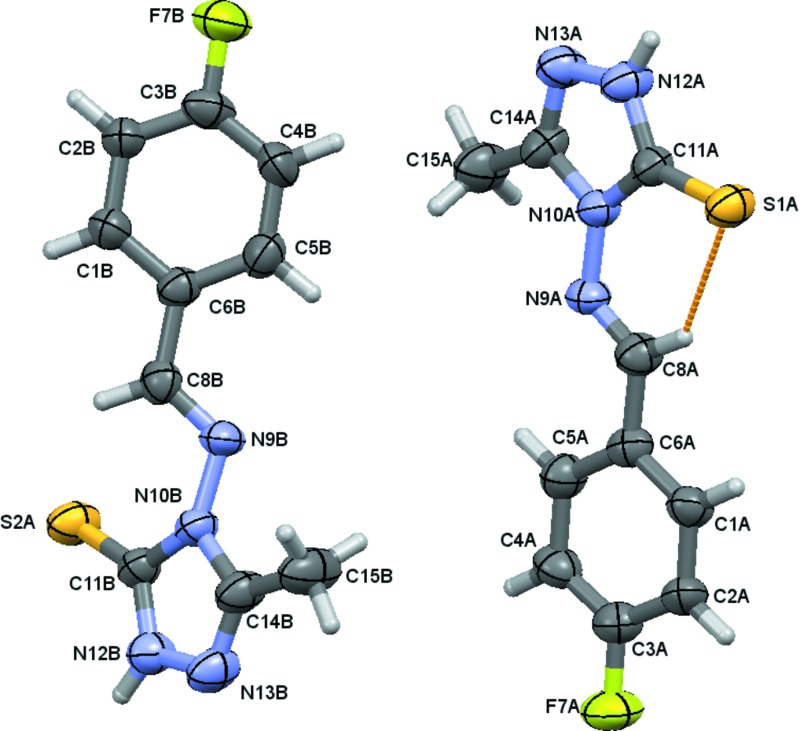
A view of the title molecule, with displacement ellipsoids drawn at the 50% probability level and an intramolecular hydrogen bond is drawn as a dashed line.

**Fig. 2.**
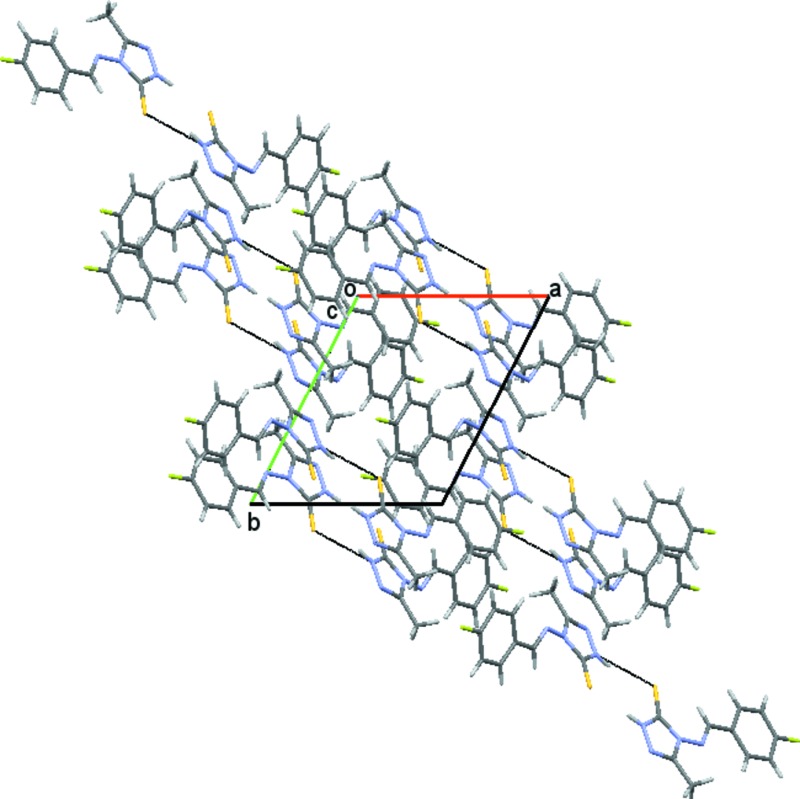
A viewed along the c axis of the crystal packing of the title compound. Hydrogen bonds are drawn as a dashed lines.

. DOI: 10.1107/S2056989015020125/hb7528fig1.tif
A view of the title mol­ecule, with displacement ellipsoids drawn at the 50% probability level and an intra­molecular hydrogen bond is drawn as a dashed line.

c . DOI: 10.1107/S2056989015020125/hb7528fig2.tif
A viewed along the *c* axis of the crystal packing of the title compound. Hydrogen bonds are drawn as a dashed lines.

**Fig. 3.**
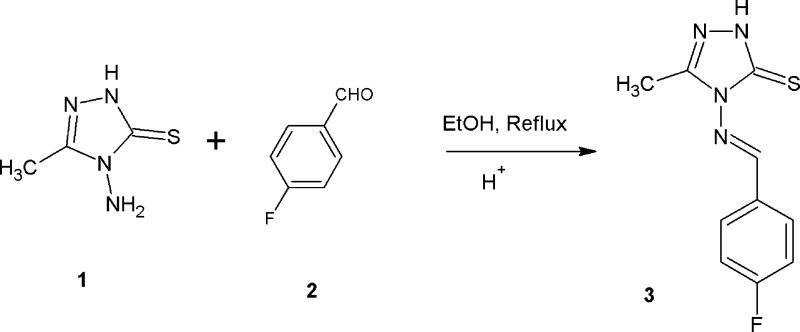
Reaction scheme.

. DOI: 10.1107/S2056989015020125/hb7528fig3.tif
Reaction scheme.

CCDC reference: 1433065

Additional supporting information:  crystallographic information; 3D view; checkCIF report

## Figures and Tables

**Table 1:** Hydrogen-bond geometry (Å, °)

*D*—H⋯*A*	*D*—H	H⋯*A*	*D*⋯*A*	*D*—H⋯*A*
C8*A*—H8*A*⋯S1*A*	0.93	2.47	3.232 (5)	139
N12*A*—H12*A*⋯S1*B* ^i^	0.86	2.54	3.391 (4)	173
N12*B*—H12*B*⋯S1*A* ^ii^	0.86	2.49	3.326 (4)	163

Symmetry codes: (i) 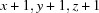; (ii) 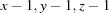.

## supplementary crystallographic information

### S1. Comment

As part of our crystal structural studies of new Schiff base derivatives useful for the preparation of biologically active 5-membered heterocyclic rings such as 3-methyl-1*H*-1,2,4-triazole-5(4*H*)-thione (Manjula *et al*., 2015). We now elucidate the crystal structure of the title molecule.

The asymmetric unit consists of two symmetry-independent molecules (A and B) of the title compound as shown in Fig. 1. The conformation of the molecules are different as evidenced by the dihedral angles [7.3 (3) ° and 41.1 (3) ° between triazole and flurobenzene moiety for molecules A and B, respectively]. An intramolecular interaction of the type C8A—H8A···S1A is observed in molecule A and is absent in molecule B (Fig. 1).

The packing of the molecules is as shown in figure 2. The molecules are linked through the N—H···S hydrogen bonds (Fig. 2) forming *R*_2_^2^(8) ring motifs. A π–π interaction exists between centroid s: *Cg*1 A···*Cg*2 A (distance = 3.739 (6) Å) and *Cg*2 A···*Cg*1 A (distance = 3.740 (6) Å). Where, *Cg*1 A is N10A/C11A/N12A/N13A/C14A.

### S2. Experimental

For the synthesis of titled compound (3), a suspension of 4-fluoro benzaldehyde (2) (0.01 mol) in ethanol (15 ml) was added to 4-Amino-3-methyl-1*H*-1,2,4-triazole-5(4*H*)-thione (1) (0.01 mol) and heated to get a clear solution (scheme). To this few drops of conc·H_2_SO_4_ were added as a catalyst and refluxed for 36hrs on water bath. The precipitate formed was filtered and recrystallized from suitable reagent to get the titled compound. Single crystals were obtained from acetic acid. (mp. 431–433 K).

### S3. Refinement

Crystal data, data collection and structure refinement details are summarized in Table 1. The hydrogen atoms were fixed geometrically (C—H = 0.93–0.96 Å, N—H = 0.98 Å) and allowed to ride on their parent atoms with *U*_iso_(H) = 1.2*U*_eq_(C/N).

### Crystal data

**Table:**

C_10_H_9_FN_4_S	*Z* = 4
*M**_r_* = 236.27	*F*(000) = 488
Triclinic, *P*1	*D*_x_ = 1.411 Mg m^−^^3^
*a* = 9.1878 (13) Å	Mo *K*α radiation, λ = 0.71075 Å
*b* = 11.0083 (16) Å	θ = 3.1–50.2°
*c* = 12.9851 (18) Å	µ = 0.28 mm^−^^1^
α = 99.526 (6)°	*T* = 273 K
β = 104.963 (13)°	Block, colourless
γ = 113.202 (19)°	0.46 × 0.27 × 0.14 mm
*V* = 1112.1 (3) Å^3^	

### Data collection

**Table:**

Rigaku Saturn724+ diffractometer	3864 independent reflections
Radiation source: Sealed tube	2129 reflections with *I* > 2σ(*I*)
Confocal monochromator	*R*_int_ = 0.042
Detector resolution: 7.111 pixels mm^-1^	θ_max_ = 25.1°, θ_min_ = 3.1°
profile data from ω scans	*h* = −10→10
Absorption correction: numerical (*NUMABS*; Rigaku 1999)	*k* = −13→13
*T*_min_ = 0.913, *T*_max_ = 0.961	*l* = −15→15
7140 measured reflections	

### Refinement

**Table:**

Refinement on *F*^2^	Primary atom site location: structure-invariant direct methods
Least-squares matrix: full	Hydrogen site location: inferred from neighbouring sites
*R*[*F*^2^ > 2σ(*F*^2^)] = 0.068	H-atom parameters constrained
*wR*(*F*^2^) = 0.241	*w* = 1/[σ^2^(*F*_o_^2^) + (0.1091*P*)^2^] where *P* = (*F*_o_^2^ + 2*F*_c_^2^)/3
*S* = 1.14	(Δ/σ)_max_ = 0.001
3864 reflections	Δρ_max_ = 0.38 e Å^−^^3^
291 parameters	Δρ_min_ = −0.39 e Å^−^^3^
0 restraints	

### Special details

**Table:**

Geometry. All e.s.d.'s (except the e.s.d. in the dihedral angle between two l.s. planes) are estimated using the full covariance matrix. The cell e.s.d.'s are taken into account individually in the estimation of e.s.d.'s in distances, angles and torsion angles; correlations between e.s.d.'s in cell parameters are only used when they are defined by crystal symmetry. An approximate (isotropic) treatment of cell e.s.d.'s is used for estimating e.s.d.'s involving l.s. planes.

### Fractional atomic coordinates and isotropic or equivalent isotropic displacement parameters (Å^2^)

**Table:**

	*x*	*y*	*z*	*U*_iso_*/*U*_eq_	
S1A	0.39859 (16)	1.12479 (13)	1.02659 (11)	0.0573 (5)	
F7A	−0.4939 (4)	0.8642 (3)	0.4693 (2)	0.0760 (10)	
N9A	−0.0027 (5)	0.8484 (4)	0.9077 (3)	0.0473 (10)	
N10A	0.1291 (4)	0.8735 (4)	1.0034 (3)	0.0432 (10)	
N12A	0.3539 (5)	0.9370 (4)	1.1424 (3)	0.0561 (12)	
H12A	0.4552	0.9845	1.1908	0.067*	
N13A	0.2420 (5)	0.8131 (4)	1.1474 (3)	0.0554 (12)	
C1A	−0.1016 (6)	1.0207 (5)	0.7051 (4)	0.0500 (13)	
H1A	−0.0018	1.1037	0.7368	0.060*	
C2A	−0.2264 (6)	1.0043 (5)	0.6090 (4)	0.0530 (13)	
H2A	−0.2120	1.0753	0.5764	0.064*	
C3A	−0.3703 (6)	0.8814 (5)	0.5640 (4)	0.0522 (13)	
C4A	−0.3998 (6)	0.7748 (5)	0.6106 (4)	0.0552 (14)	
H4A	−0.5011	0.6931	0.5785	0.066*	
C5A	−0.2734 (6)	0.7923 (5)	0.7079 (4)	0.0536 (14)	
H5A	−0.2902	0.7215	0.7409	0.064*	
C6A	−0.1226 (6)	0.9155 (5)	0.7551 (4)	0.0454 (12)	
C8A	0.0114 (6)	0.9380 (5)	0.8561 (4)	0.0510 (13)	
H8A	0.1110	1.0211	0.8837	0.061*	
C11A	0.2945 (6)	0.9791 (5)	1.0568 (4)	0.0446 (12)	
C14A	0.1047 (6)	0.7753 (5)	1.0609 (4)	0.0526 (13)	
C15A	−0.0608 (6)	0.6508 (5)	1.0301 (4)	0.0719 (18)	
H15A	−0.1473	0.6790	1.0294	0.108*	
H15B	−0.0529	0.6011	1.0838	0.108*	
H15C	−0.0889	0.5918	0.9572	0.108*	
S1B	−0.26267 (16)	0.12273 (14)	0.35120 (11)	0.0610 (5)	
F7B	0.5646 (4)	0.4091 (3)	0.9385 (2)	0.0814 (11)	
N9B	0.1189 (5)	0.4053 (4)	0.4714 (3)	0.0496 (11)	
N10B	0.0075 (5)	0.3641 (4)	0.3610 (3)	0.0484 (10)	
N12B	−0.2067 (5)	0.2789 (4)	0.2102 (3)	0.0521 (11)	
H12B	−0.3036	0.2243	0.1587	0.063*	
N13B	−0.0935 (5)	0.3990 (4)	0.1997 (4)	0.0613 (13)	
C1B	0.2667 (6)	0.2306 (5)	0.6557 (4)	0.0508 (13)	
H1B	0.2079	0.1415	0.6058	0.061*	
C2B	0.3733 (6)	0.2533 (5)	0.7627 (4)	0.0512 (13)	
H2B	0.3869	0.1811	0.7849	0.061*	
C3B	0.4578 (6)	0.3861 (5)	0.8343 (4)	0.0562 (14)	
C4B	0.4449 (7)	0.4946 (5)	0.8038 (4)	0.0625 (15)	
H4B	0.5082	0.5838	0.8534	0.075*	
C5B	0.3360 (6)	0.4708 (5)	0.6976 (4)	0.0555 (14)	
H5B	0.3230	0.5439	0.6768	0.067*	
C6B	0.2462 (6)	0.3377 (5)	0.6221 (4)	0.0451 (12)	
C8B	0.1354 (6)	0.3082 (5)	0.5079 (4)	0.0475 (12)	
H8B	0.0759	0.2175	0.4608	0.057*	
C11B	−0.1542 (6)	0.2535 (5)	0.3070 (4)	0.0470 (12)	
C14B	0.0355 (6)	0.4495 (5)	0.2934 (4)	0.0574 (14)	
C15B	0.1950 (7)	0.5814 (6)	0.3281 (5)	0.087 (2)	
H15D	0.2052	0.6437	0.3938	0.130*	
H15E	0.1922	0.6232	0.2688	0.130*	
H15F	0.2904	0.5622	0.3439	0.130*	

### Atomic displacement parameters (Å^2^)

**Table:**

	*U*^11^	*U*^22^	*U*^33^	*U*^12^	*U*^13^	*U*^23^
S1A	0.0503 (8)	0.0540 (8)	0.0611 (10)	0.0180 (7)	0.0128 (7)	0.0283 (7)
F7A	0.065 (2)	0.094 (2)	0.061 (2)	0.0378 (18)	0.0029 (16)	0.0320 (18)
N9A	0.044 (2)	0.050 (2)	0.041 (2)	0.021 (2)	0.0039 (19)	0.015 (2)
N10A	0.044 (2)	0.048 (2)	0.038 (2)	0.022 (2)	0.0117 (19)	0.0175 (19)
N12A	0.049 (3)	0.062 (3)	0.057 (3)	0.022 (2)	0.016 (2)	0.032 (2)
N13A	0.054 (3)	0.056 (3)	0.050 (3)	0.018 (2)	0.014 (2)	0.024 (2)
C1A	0.050 (3)	0.051 (3)	0.041 (3)	0.016 (2)	0.012 (2)	0.016 (2)
C2A	0.064 (3)	0.052 (3)	0.039 (3)	0.025 (3)	0.011 (3)	0.018 (3)
C3A	0.050 (3)	0.061 (3)	0.047 (3)	0.027 (3)	0.014 (3)	0.020 (3)
C4A	0.043 (3)	0.059 (3)	0.049 (3)	0.019 (3)	0.005 (2)	0.010 (3)
C5A	0.050 (3)	0.048 (3)	0.056 (3)	0.015 (3)	0.016 (3)	0.022 (3)
C6A	0.045 (3)	0.057 (3)	0.043 (3)	0.029 (3)	0.019 (2)	0.019 (3)
C8A	0.044 (3)	0.053 (3)	0.053 (3)	0.020 (2)	0.015 (2)	0.020 (3)
C11A	0.046 (3)	0.045 (3)	0.046 (3)	0.023 (2)	0.015 (2)	0.018 (2)
C14A	0.053 (3)	0.054 (3)	0.061 (4)	0.029 (3)	0.022 (3)	0.030 (3)
C15A	0.066 (4)	0.064 (4)	0.073 (4)	0.015 (3)	0.020 (3)	0.037 (3)
S1B	0.0540 (9)	0.0608 (9)	0.0584 (10)	0.0150 (7)	0.0144 (7)	0.0318 (7)
F7B	0.085 (2)	0.078 (2)	0.050 (2)	0.0233 (18)	−0.0030 (17)	0.0199 (17)
N9B	0.050 (2)	0.047 (2)	0.036 (2)	0.015 (2)	0.0017 (19)	0.015 (2)
N10B	0.051 (2)	0.042 (2)	0.043 (2)	0.016 (2)	0.008 (2)	0.019 (2)
N12B	0.044 (2)	0.052 (2)	0.051 (3)	0.014 (2)	0.011 (2)	0.022 (2)
N13B	0.055 (3)	0.058 (3)	0.067 (3)	0.017 (2)	0.017 (2)	0.037 (2)
C1B	0.058 (3)	0.046 (3)	0.041 (3)	0.021 (2)	0.013 (2)	0.012 (2)
C2B	0.060 (3)	0.045 (3)	0.042 (3)	0.023 (3)	0.011 (3)	0.013 (3)
C3B	0.052 (3)	0.064 (4)	0.038 (3)	0.014 (3)	0.011 (2)	0.020 (3)
C4B	0.074 (4)	0.042 (3)	0.045 (3)	0.016 (3)	0.005 (3)	0.004 (3)
C5B	0.070 (4)	0.048 (3)	0.058 (4)	0.032 (3)	0.026 (3)	0.023 (3)
C6B	0.045 (3)	0.045 (3)	0.047 (3)	0.019 (2)	0.016 (2)	0.021 (2)
C8B	0.052 (3)	0.047 (3)	0.050 (3)	0.027 (2)	0.020 (2)	0.014 (3)
C11B	0.052 (3)	0.045 (3)	0.043 (3)	0.020 (2)	0.015 (2)	0.020 (2)
C14B	0.052 (3)	0.054 (3)	0.062 (4)	0.018 (3)	0.015 (3)	0.032 (3)
C15B	0.067 (4)	0.071 (4)	0.083 (5)	0.001 (3)	0.002 (3)	0.047 (3)

### Geometric parameters (Å, º)

**Table:**

S1A—C11A	1.683 (5)	S1B—C11B	1.684 (4)
F7A—C3A	1.367 (5)	F7B—C3B	1.365 (5)
N9A—N10A	1.390 (5)	N9B—N10B	1.404 (5)
N9A—C8A	1.266 (5)	N9B—C8B	1.283 (5)
N10A—C11A	1.393 (5)	N10B—C11B	1.391 (5)
N10A—C14A	1.392 (5)	N10B—C14B	1.386 (6)
N12A—H12A	0.8600	N12B—H12B	0.8600
N12A—N13A	1.371 (5)	N12B—N13B	1.377 (5)
N12A—C11A	1.344 (5)	N12B—C11B	1.341 (5)
N13A—C14A	1.314 (6)	N13B—C14B	1.307 (6)
C1A—H1A	0.9300	C1B—H1B	0.9300
C1A—C2A	1.389 (6)	C1B—C2B	1.393 (6)
C1A—C6A	1.391 (6)	C1B—C6B	1.386 (6)
C2A—H2A	0.9300	C2B—H2B	0.9300
C2A—C3A	1.360 (6)	C2B—C3B	1.374 (6)
C3A—C4A	1.373 (6)	C3B—C4B	1.356 (7)
C4A—H4A	0.9300	C4B—H4B	0.9300
C4A—C5A	1.402 (6)	C4B—C5B	1.391 (6)
C5A—H5A	0.9300	C5B—H5B	0.9300
C5A—C6A	1.393 (6)	C5B—C6B	1.393 (6)
C6A—C8A	1.454 (6)	C6B—C8B	1.464 (6)
C8A—H8A	0.9300	C8B—H8B	0.9300
C14A—C15A	1.488 (6)	C14B—C15B	1.491 (6)
C15A—H15A	0.9600	C15B—H15D	0.9600
C15A—H15B	0.9600	C15B—H15E	0.9600
C15A—H15C	0.9600	C15B—H15F	0.9600
			
C8A—N9A—N10A	120.5 (4)	C8B—N9B—N10B	116.0 (4)
N9A—N10A—C11A	133.3 (4)	C11B—N10B—N9B	129.7 (4)
N9A—N10A—C14A	118.5 (4)	C14B—N10B—N9B	121.4 (4)
C14A—N10A—C11A	108.1 (4)	C14B—N10B—C11B	108.0 (4)
N13A—N12A—H12A	122.3	N13B—N12B—H12B	122.5
C11A—N12A—H12A	122.3	C11B—N12B—H12B	122.5
C11A—N12A—N13A	115.4 (4)	C11B—N12B—N13B	114.9 (4)
C14A—N13A—N12A	103.5 (4)	C14B—N13B—N12B	103.4 (4)
C2A—C1A—H1A	119.3	C2B—C1B—H1B	119.3
C2A—C1A—C6A	121.4 (4)	C6B—C1B—H1B	119.3
C6A—C1A—H1A	119.3	C6B—C1B—C2B	121.5 (5)
C1A—C2A—H2A	121.0	C1B—C2B—H2B	121.2
C3A—C2A—C1A	118.1 (4)	C3B—C2B—C1B	117.6 (4)
C3A—C2A—H2A	121.0	C3B—C2B—H2B	121.2
F7A—C3A—C4A	118.2 (4)	F7B—C3B—C2B	117.7 (4)
C2A—C3A—F7A	118.5 (4)	C4B—C3B—F7B	119.4 (5)
C2A—C3A—C4A	123.3 (5)	C4B—C3B—C2B	122.8 (5)
C3A—C4A—H4A	120.8	C3B—C4B—H4B	120.4
C3A—C4A—C5A	118.3 (5)	C3B—C4B—C5B	119.2 (5)
C5A—C4A—H4A	120.8	C5B—C4B—H4B	120.4
C4A—C5A—H5A	119.9	C4B—C5B—H5B	119.9
C6A—C5A—C4A	120.2 (4)	C4B—C5B—C6B	120.2 (4)
C6A—C5A—H5A	119.9	C6B—C5B—H5B	119.9
C1A—C6A—C5A	118.8 (4)	C1B—C6B—C5B	118.6 (4)
C1A—C6A—C8A	119.3 (4)	C1B—C6B—C8B	119.1 (4)
C5A—C6A—C8A	121.9 (4)	C5B—C6B—C8B	122.3 (4)
N9A—C8A—C6A	122.3 (4)	N9B—C8B—C6B	121.2 (4)
N9A—C8A—H8A	118.9	N9B—C8B—H8B	119.4
C6A—C8A—H8A	118.9	C6B—C8B—H8B	119.4
N10A—C11A—S1A	130.5 (3)	N10B—C11B—S1B	129.7 (3)
N12A—C11A—S1A	127.4 (4)	N12B—C11B—S1B	127.8 (4)
N12A—C11A—N10A	102.2 (4)	N12B—C11B—N10B	102.4 (4)
N10A—C14A—C15A	122.8 (4)	N10B—C14B—C15B	122.2 (4)
N13A—C14A—N10A	110.9 (4)	N13B—C14B—N10B	111.2 (4)
N13A—C14A—C15A	126.2 (4)	N13B—C14B—C15B	126.6 (4)
C14A—C15A—H15A	109.5	C14B—C15B—H15D	109.5
C14A—C15A—H15B	109.5	C14B—C15B—H15E	109.5
C14A—C15A—H15C	109.5	C14B—C15B—H15F	109.5
H15A—C15A—H15B	109.5	H15D—C15B—H15E	109.5
H15A—C15A—H15C	109.5	H15D—C15B—H15F	109.5
H15B—C15A—H15C	109.5	H15E—C15B—H15F	109.5
			
F7A—C3A—C4A—C5A	−179.9 (4)	F7B—C3B—C4B—C5B	180.0 (4)
N9A—N10A—C11A—S1A	2.6 (8)	N9B—N10B—C11B—S1B	−7.1 (8)
N9A—N10A—C11A—N12A	−178.2 (4)	N9B—N10B—C11B—N12B	171.0 (4)
N9A—N10A—C14A—N13A	178.6 (4)	N9B—N10B—C14B—N13B	−171.9 (4)
N9A—N10A—C14A—C15A	−5.3 (7)	N9B—N10B—C14B—C15B	7.9 (8)
N10A—N9A—C8A—C6A	179.4 (4)	N10B—N9B—C8B—C6B	−179.2 (4)
N12A—N13A—C14A—N10A	−0.4 (5)	N12B—N13B—C14B—N10B	0.9 (6)
N12A—N13A—C14A—C15A	−176.4 (5)	N12B—N13B—C14B—C15B	−178.9 (6)
N13A—N12A—C11A—S1A	−179.8 (3)	N13B—N12B—C11B—S1B	176.2 (4)
N13A—N12A—C11A—N10A	0.9 (5)	N13B—N12B—C11B—N10B	−1.9 (5)
C1A—C2A—C3A—F7A	−179.9 (5)	C1B—C2B—C3B—F7B	−178.9 (4)
C1A—C2A—C3A—C4A	1.8 (8)	C1B—C2B—C3B—C4B	−2.0 (8)
C1A—C6A—C8A—N9A	177.3 (5)	C1B—C6B—C8B—N9B	−176.7 (5)
C2A—C1A—C6A—C5A	−0.8 (7)	C2B—C1B—C6B—C5B	0.4 (8)
C2A—C1A—C6A—C8A	−179.6 (5)	C2B—C1B—C6B—C8B	178.6 (4)
C2A—C3A—C4A—C5A	−1.7 (8)	C2B—C3B—C4B—C5B	3.1 (9)
C3A—C4A—C5A—C6A	0.2 (8)	C3B—C4B—C5B—C6B	−2.4 (8)
C4A—C5A—C6A—C1A	1.0 (7)	C4B—C5B—C6B—C1B	0.7 (8)
C4A—C5A—C6A—C8A	179.8 (5)	C4B—C5B—C6B—C8B	−177.4 (5)
C5A—C6A—C8A—N9A	−1.4 (8)	C5B—C6B—C8B—N9B	1.4 (7)
C6A—C1A—C2A—C3A	−0.5 (8)	C6B—C1B—C2B—C3B	0.2 (8)
C8A—N9A—N10A—C11A	−6.6 (8)	C8B—N9B—N10B—C11B	45.3 (7)
C8A—N9A—N10A—C14A	176.6 (4)	C8B—N9B—N10B—C14B	−147.4 (5)
C11A—N10A—C14A—N13A	1.0 (6)	C11B—N10B—C14B—N13B	−2.1 (6)
C11A—N10A—C14A—C15A	177.1 (5)	C11B—N10B—C14B—C15B	177.7 (5)
C11A—N12A—N13A—C14A	−0.3 (6)	C11B—N12B—N13B—C14B	0.7 (6)
C14A—N10A—C11A—S1A	179.7 (4)	C14B—N10B—C11B—S1B	−175.8 (4)
C14A—N10A—C11A—N12A	−1.1 (5)	C14B—N10B—C11B—N12B	2.3 (5)

### Hydrogen-bond geometry (Å, º)

**Table:**

*D*—H···*A*	*D*—H	H···*A*	*D*···*A*	*D*—H···*A*
C8*A*—H8*A*···S1*A*	0.93	2.47	3.232 (5)	139
N12*A*—H12*A*···S1*B*^i^	0.86	2.54	3.391 (4)	173
N12*B*—H12*B*···S1*A*^ii^	0.86	2.49	3.326 (4)	163

Symmetry codes: (i) *x*+1, *y*+1, *z*+1; (ii) *x*−1, *y*−1, *z*−1.
