# Supplementary figures and images for: Crystal structure of 4-[(E)-(4-fluoro­benzyl­idene)amino]-3-methyl-1H-1,2,4-triazole-5(4H)-thione
Source: Acta Crystallogr E Crystallogr Commun. 2015 Nov 4;71(Pt 12):o912–3. doi: 10.1107/S2056989015020125 (PMC4719876; doi:10.1107/S2056989015020125)

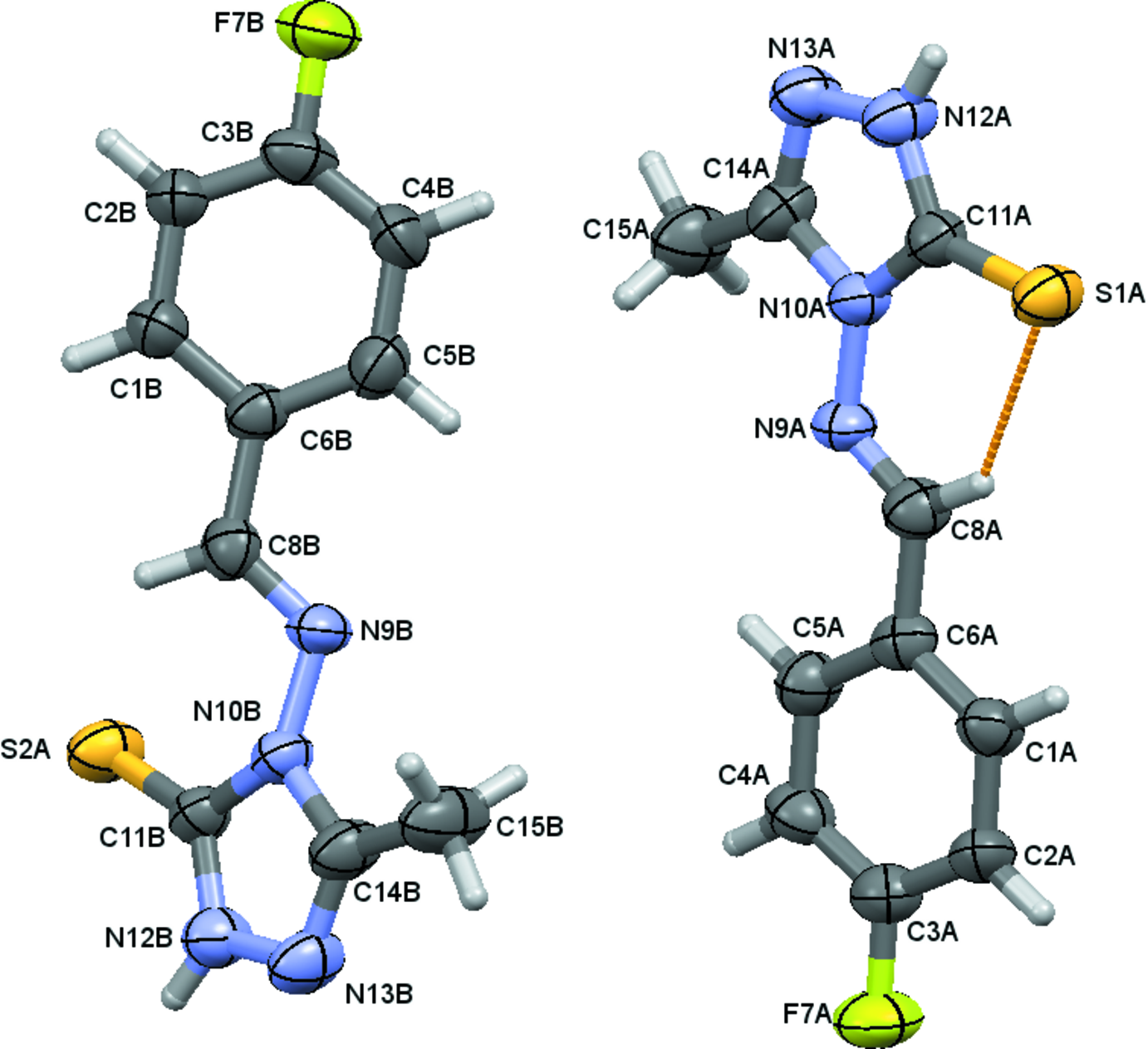

Supplement: Supplementary file 4 [file e-71-0o912-fig1.tif]

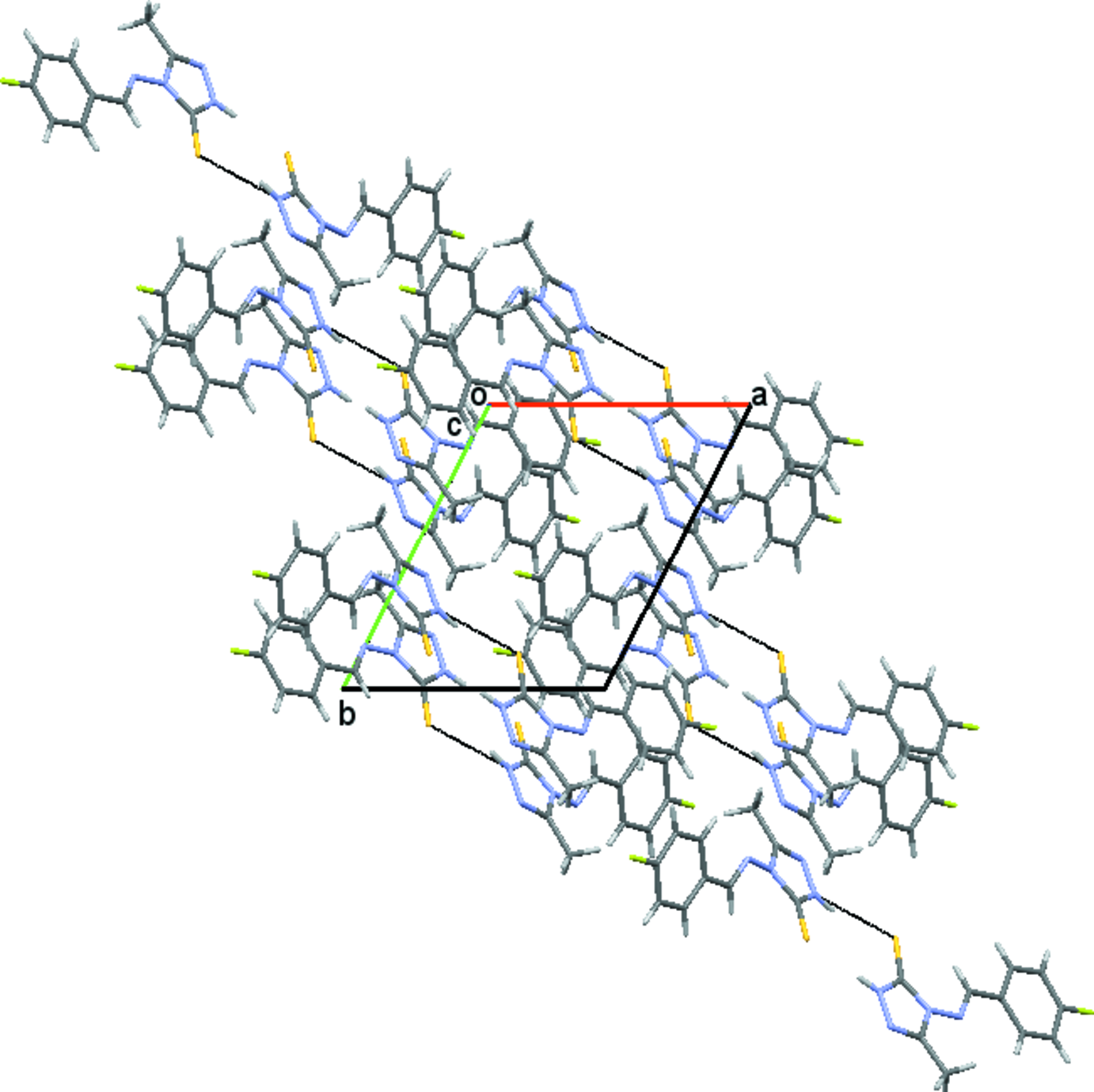

Supplement: Supplementary file 5 [file e-71-0o912-fig2.tif]
